# The Dynamin Chemical Inhibitor Dynasore Impairs Cholesterol Trafficking and Sterol-Sensitive Genes Transcription in Human HeLa Cells and Macrophages

**DOI:** 10.1371/journal.pone.0029042

**Published:** 2011-12-19

**Authors:** Emmanuelle Girard, Jean Louis Paul, Natalie Fournier, Philippe Beaune, Ludger Johannes, Christophe Lamaze, Benoît Védie

**Affiliations:** 1 AP-HP (Assistance Publique – Hôpitaux de Paris), Hôpital Européen Georges Pompidou, Service de Biochimie, Paris, France; 2 Université Paris-Sud, EA 4529, UFR de Pharmacie, Châtenay-Malabry, France; 3 CNRS UMR144, Paris, France; 4 Institut Curie, Centre de Recherche, Laboratoire Trafic, Signalisation et Ciblage Intracellulaires, Paris, France; Institute of Molecular and Cell Biology, Singapore

## Abstract

Intracellular transport of cholesterol contributes to the regulation of cellular cholesterol homeostasis by mechanisms that are yet poorly defined. In this study, we characterized the impact of dynasore, a recently described drug that specifically inhibits the enzymatic activity of dynamin, a GTPase regulating receptor endocytosis and cholesterol trafficking. Dynasore strongly inhibited the uptake of low-density lipoprotein (LDL) in HeLa cells, and to a lower extent in human macrophages. In both cell types, dynasore treatment led to the abnormal accumulation of LDL and free cholesterol (FC) within the endolysosomal network. The measure of cholesterol esters (CE) further showed that the delivery of regulatory cholesterol to the endoplasmic reticulum (ER) was deficient. This resulted in the inhibition of the transcriptional control of the three major sterol-sensitive genes, sterol-regulatory element binding protein 2 (SREBP-2), 3-hydroxy-3-methyl-coenzymeA reductase (HMGCoAR), and low-density lipoprotein receptor (LDLR). The sequestration of cholesterol in the endolysosomal compartment impaired both the active and passive cholesterol efflux in HMDM. Our data further illustrate the importance of membrane trafficking in cholesterol homeostasis and validate dynasore as a new pharmacological tool to study the intracellular transport of cholesterol.

## Introduction

Membrane trafficking contributes to cell cholesterol homeostasis through the control of intracellular cholesterol levels and compartmentalization. Little is known about the transport pathways involved in cholesterol trafficking and the associated molecular machinery [1]. The level of cellular cholesterol results from the tight control of both cholesterol neosynthesis and low-density lipoprotein (LDL)-derived cholesterol uptake. The uptake of LDL-derived cholesterol at the plasma membrane occurs primarily through clathrin-dependent endocytosis of the low-density lipoprotein receptor (LDLR), as shown in the seminal studies by Brown and Goldstein [2]. This process delivers lipoprotein-associated cholesterol esters (CE) first to the early endosome (EE) and then to the late endosomal/lysosomal network. At this stage, the acid hydrolases de-esterify CE into free cholesterol (FC), which will leave the endolysosomal network to reach the endoplasmic reticulum (ER) either directly or indirectly after transiting through the Golgi apparatus and the plasma membrane [3]. ER membranes, which are characterized by a poor content in cholesterol, are home to the principal effectors of cellular cholesterol homeostasis. Key of this regulation is a family of membrane-bound transcription factors, sterol regulatory element binding proteins (SREBPs). Under low sterol concentration, SREBP is transported to the Golgi complex where it is activated by proteolytic processing. This cleavage releases the active form of SREBP, which is translocated to the nucleus where it regulates the transcription of sterol responsive genes involved in cholesterol synthesis (3-hydroxy-3-methyl-coenzymeA reductase (HMGCoAR)) or uptake (LDLR) [4]. Cholesterol transport to the ER is therefore a key element of the cholesterol homeostasis machinery. The levels of cholesterol are constantly monitored by the two chaperone proteins SCAP (SREBP cleavage activating protein) and INSIG (insulin-induced gene protein). SCAP is a SREBP Golgi escort protein while INSIG is an ER anchored protein. Binding of cholesterol or oxysterols to SCAP or to INSIG, respectively, promotes the interaction between SCAP and INSIG. The formation of this complex masks the binding site on SCAP that is recognized by the vesicular coat transport complex COPII, thereby blocking SREBP transport from the ER to the Golgi apparatus and the release of the cleaved SREBP active form [5], [6]. Another important actor of this tight regulation is the acyl CoA:cholesterol acyltransferase (ACAT) enzyme localized in the ER [7]. ACAT rapidly esterifies FC in CE to be stored in cytoplasmic lipid droplets, in response to an increased concentration of cholesterol in the ER. ACAT activity is therefore required for decreasing the amount of cytotoxic FC and for maintaining a low level of cholesterol in the ER membranes, such that minimal variations of sterol concentrations can be sensed. In some cases, ER-derived vesicular structures that are positive for ACAT can also be involved in this process. It has been proposed that these structures, which are distinct from the conventional ER, may protect the non-fragmented ER from being overloaded with cholesterol [8].

The GTPase dynamin is a mechano-chemical enzyme required for the pinching and release of a completed clathrin-coated pit from the plasma membrane. In HeLa cells expressing the K44A inactivated form of dynamin, we have revealed a new role for dynamin in the delivery of endolysosomal FC to the ER [9]. The inactivation of dynamin led to a Niemann-Pick type C-like phenotype (NPC) with the accumulation of swollen late endosomes/lysosomes (LE/LS) engorged with FC. Dynamin inactivation was also associated with a strong reduction of sterol-sensitive genes regulation and a decrease of the esterification of the intracellular pool of cholesterol by ACAT. If K44A HeLa cells are an easily amenable cell model, we sought to study the role of dynamin in more physiologically relevant cell types such as macrophages, which play a key role in atherosclerosis. However there are technical limitations to obtain macrophages expressing the inactivated form of dynamin. We therefore took advantage of the membrane permeant chemical compound, dynasore, a recently described noncompetitive inhibitor of the GTPase activity of dynamin [10], [11]. Dynasore presents also the advantage to act within minutes and to have a completely reversible activity. To validate the use of this new drug, we studied the impact of dynasore on critical steps of cholesterol trafficking in both HeLa cells and HMDM. We show here that dynasore rapidly inhibits the egress of free cholesterol from the endolysosomal network in these two cell types. As a result, the sterol-sensitive gene regulation of cholesterol homeostasis is inhibited. Dynasore is therefore a new drug that will be useful for further understanding cholesterol endosomal trafficking, a key step in cholesterol homeostasis.

## Materials and Methods

### Cells and reagents

HeLa cells were cultured in Dulbecco modified Eagle's medium (DMEM) with 10% heat-inactivated fetal calf serum (FCS) (Invitrogen, Cergy-Pontoise, France), 2 mM glutamine, 100 UI/ml penicillin, 100 µg/ml streptomycin (Sigma-Aldrich, St Louis, MO). Cells were treated with dynasore (Sigma-Aldrich) at 80 µM in the presence of 1% lipoprotein-deficient serum (LPDS) medium to avoid inactivation of dynasore by serum proteins. U18666A (Sigma-Aldrich) was used at 3 µg/ml in 1% LPDS medium.

Monocytes were isolated from human blood of normolipidemic donors by ficoll density gradient (Eurobio, les Ulis, France) and subsequently differentiated into human monocytes-derived macrophages (HMDM) by adhesion on plastic Primaria plates (Falcon BD, Franklin Lakes, NJ,) for 7 days in RPM1640 medium, supplemented with 10% heat-inactived FCS, 2 mM glutamine, 100 UI/ml penicillin, 100 µg/ml streptomycin, and 20 ng/ml hM-CSF (human macrophages colony-stimulating factor) (AbCys, Paris, France).

HeLa cells expressing a GFP tagged M6PR were described in [12]. Cells were cultured in Dulbecco modified Eagle's medium (DMEM) with 10% heat-inactivated fetal calf serum (FCS) (Invitrogen,), 2 mM glutamine, 100 UI/ml penicillin, 100 µg/ml streptomycin (Sigma-Aldrich,) completed with geneticin. They were treated with dynasore the same way that HeLa cells.

### Antibodies

The mouse anti-human Lamp-1 antibody was from BD Transduction Laboratories. Primary antibodies were revealed by incubation with Alexa Fluor®488 donkey anti-mouse IgG (Invitrogen).

### Cholesterol delivery to the cell

Human LDL (d = 1.019–1.063 g/ml) were separated from fresh plasma by sequential ultracentrifugation as described previously [9]. HeLa cells deprived of sterols for 48 h in 10% LPDS medium were incubated for the indicated times with LDL (200 µg/ml), supplemented with 50 µM pravastatin and 50 µM sodium mevalonate. HMDM were incubated with 50 or 100 µg/ml of acetylated LDL (AcLDL) prepared using the standard acetic anhydride-sodium acetate solution on ice as described [13].

### LDL and AcLDL labeling

LDL were labeled with DiI (1,1′-dioctadecyl-3,3,3′3′-tetramethyl-indocarbocyanine) as described previously [14]. Briefly, DiI were added to LDL to a final ratio of 300 µg for 1 mg LDL protein. After incubation for 18 h at 37°C under nitrogen and light protection, DiI-labeled LDL were isolated by ultracentrifugation and extensively dialyzed at 4°C against PBS (Eurobio). For DiI-AcLDL production, AcLDL were first labeled with DiI and acetylated.

### LDL uptake assay

LDL uptake was described in [9]. Briefly, cells grown in LPDS medium during 48 h were washed in PBS and incubated at 37°C with increasing concentrations of DiI-LDL (HeLa cells) or DiI-AcLDL (HMDM) as indicated. After 4 h, cells were treated by trypsin to remove cell surface bound fluorescent LDL. Cells were detached and washed twice in PBS at 4°C. The fluorescence of internalized DiI-LDL or DiI-AcLDL was measured by flow cytometry (emission at 585 nm) and expressed as mean fluorescence intensity.

### Measurement of cellular cholesterol

After 48 h of culture in LPDS medium, cells were incubated for 6 h with 200 µg/ml LDL (HeLa cells) or 50 µg/ml AcLDL (HMDM) and then lysed in 0.2 M NaOH. Total cholesterol was extracted with methanol (2.5 ml) followed by hexane (5 ml). About 4.5 ml of the hexane phase was evaporated under vacuum and dissolved in mobile phase. Separation of FC and CE including cholesteryl docohexanoate (CDH), cholesteryl arachidonate (CA), cholesteryl linoleate (CL), cholesteryl myristate (CM), cholesteryl oleate (CO) and cholesteryl stearate (CS) was done by reverse phase HPLC on a C-18 column (25×0.46 cm length, 5-µm pore size, Sigma-Aldrich) by measuring the 205 nm absorbance after elution with acetonitrile/isopropanol (30/70, v/v) [15], [16]. The amount of proteins present in cell lysates and LDL preparations was measured using the bicinchoninic acid (BCA) method [17].

### ACAT activity

120 µM sodium myristate (Sigma-Aldrich) was added during cholesterol delivery to the cells. The ACAT enzyme activity was evaluated by quantifying the incorporation of myristate into cholesteryl myristate by HPLC as described above. The ACAT inhibitor Sandoz 58-035 (Sigma-Aldrich) (10 µg/ml) was added to the cholesterol loading medium. When ACAT activity is inhibited, CE are provided by pre-existing pools such as endocytosed LDL. Therefore, the difference in cholesterol esterification measured by HPLC with and without Sandoz 58-035 represents the specific amount of cholesterol esterified by ACAT.

### Cellular cholesterol mass efflux to apoA-I and HDL in cholesterol-loaded macrophages

After treatment with dynasore or U18666A, HMDM were equilibrated in RPMI1640 0.2% BSA for 16 h in the presence or not of 5 µg/ml 22-hydroxy-cholesterol (22OH-C) and 10 µM 9-cis retinoic acid (9cRA) (Sigma-Aldrich). Cellular cholesterol efflux to either 1 mg/ml methyl-β-cyclodextrin (MβCD), 10 µg/ml lipid-free apoA-I (Sigma-Aldrich) or 15 µg/ml high-density lipoprotein-phospholipids (HDL-PL isolated from normolipidemic human plasma by preparative ultracentrifugation) was measured during a 4 h chase period. Drugs were maintained during the equilibration and efflux periods. At the end of the efflux, the medium was collected and the cells lysed in 0.2 M NaOH. Cell and media were extracted and analyzed for free and esterified cholesterol mass by HPLC as described above. HDL samples were separately analyzed to allow correction for HDL cholesterol present in relevant media samples. Mass cholesterol efflux is expressed as the percentage of efflux (medium cholesterol over total cholesterol-medium and cells) [18].

### Immunofluorescence

Analysis of LDL-derived cholesterol trafficking was performed by immunofluorescence as described [9]. Briefly, HeLa cells and HMDM were grown on cover slips for 48 h in LPDS medium. To monitor LDL trafficking, cells were incubated with fluorescent LDL for indicated times. Cells were incubated with 200 µg/ml LDL or 50 µg/ml AcLDL and then stained with filipin (50 µg/ml) to detect FC as described [19]. Cells were fixed in 4% paraformaldehyde (PFA) in PBS. For immunolocalization studies, cells were incubated with primary and secondary antibodies diluted in PBS/BSA with saponin. Cover slips were mounted in MOWIOL, and cells were imaged with an epifluorescent Leica microscope.

### RNA extraction, RT-PCR and real time quantitative PCR

Total RNA was extracted from HeLa cells using the RNeasy Mini kit (Qiagen, Courtaboeuf, France). One microgram of total RNA was transcribed to cDNA using random hexamers (Amersham, Orsay, France) and SuperScriptII reverse transcriptase (Invitrogen). Specific primers were described previously [9]. Real-time quantitative PCR analyses were performed using the Sybr® Green reagents kit (Applied Biosystems, Courtaboeuf, France) with an ABI PRISM 7900HT Sequence detector instrument (Applied Biosystems) according to the manufacturer's instructions. Amplification was carried out in a final volume of 10 µL with 20 ng of reverse transcribed total RNA, 150 nM (HMGCoAR, LDLR) or 300 nM (UBC and SREBF-2) of both sense and antisense primers (Eurogentec, Liège, Belgium) in the Sybr® Green PCR Master Mix (MESA GREEN qPCR, Eurogentec). The cycling conditions comprised 40 cycles at 95°C for 15 s and 60°C for 1 min. Relative quantification for a given gene, expressed as fold-variation over control at T0, was calculated after normalization to a reference gene (UBC) and determination of the CT (cycle threshold) difference between conditioned cells and control cells at T0 using the comparative CT method [20]. Efficiencies of the target and control amplification were very similar.

### Dynamin 2 RNAi Treatment

Non targeting siRNA (si scramble) and siRNA against dynamin2 (si Dyn2) (GGA CAU GAU CCU GCA GUUdTdT) were described in [21]. Briefly, HeLa cells were transfected using RNAiMAX (Invitrogen) according to the manufacturer's instructions. Knockdown of Dyn2 was observed 48 h post-treatment. Cells transfected with scramble siRNA were used as a control.

### Western Blotting

Cells were lysed in 1% triton ×100 in PBS containing protease inhibitors. Proteins (30 µg per lane) were separated on a 3–8% NuPAGE in Tris acetate gels (Invitrogen) and transferred to a PDVF membrane (Bio-Rad, Hercules, CA). The membrane was incubated for 1 h in blocking solution (5% nonfat dry milk, 0.1% Tween 20 in PBS). Dynamin 2 was detected using mouse monoclonal anti-dynamin 2 (Invitrogen). A rabbit anti-β-actin polyclonal antibody was used as loading control (Sigma). Development was performed with chemiluminescent detection kit (ECL, Amersham Life Science).

## Results

### Dynasore inhibits the uptake of LDL cholesterol in HeLa cells and human macrophages

**Figure 1:**
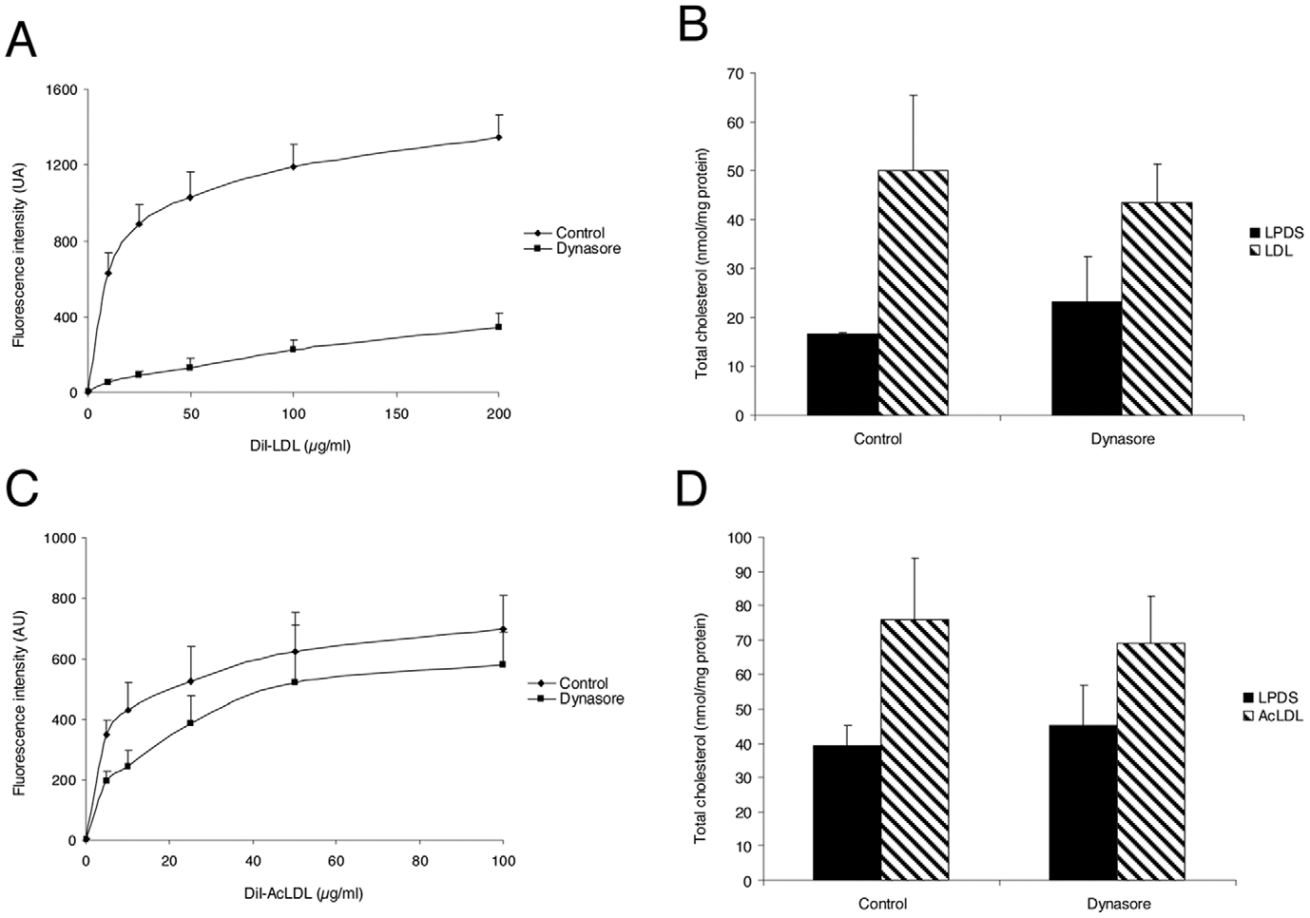
Effect of dynasore on LDL uptake and total cholesterol in HeLa cells and HMDM. Cells were incubated for 4 h with 0–200 µg/ml DiI-LDL (A) or 0–100 µg/ml DiI-AcLDL (C) at 37°C with 0.4% v/v DMSO (control) or 80 µM dynasore. The total amount of endocytosed DiI-LDL or DiI-AcLDL was measured by flow cytometry. Values represent the mean ± SD of triplicate experiments. Total cholesterol was quantified in HeLa cells (B) and HMDM (D) after 4 h of LDL uptake with 0.4% v/v DMSO (control) or 80 µM dynasore. Each value is the mean ± SD of triplicate experiments and expressed as nanomoles per mg of cell proteins.

We tested the effect of dynasore on HeLa cells, where dynamin was first reported to control CT trafficking [9]. Dynasore was tested at 80 µM, the concentration shown to efficiently block the internalization of the transferrin receptor [10]. We measured the uptake of fluorescently labeled human LDL by flow cytometry. Dynasore treatment led to a strong inhibition of LDL uptake at 10 µg/ml with less than 10% of the uptake level measured in untreated cells (Figure 1A). For comparison, the uptake of LDL was decreased by about 50% in our previous study with HeLa cells expressing the K44A inhibitory mutant of dynamin [9]. The continuous uptake of LDL for 6 hours in sterol-starved cells led to a 2.5 fold increase of the total amount of cellular cholesterol – free and esterified – as measured by HPLC in control cells. Despite the strong inhibition of LDL uptake, there was still an increase albeit more moderate in dynasore-treated cells (50±15 and 43±8 nmol/mg cell protein, respectively; Figure 1B). This is in agreement with our findings in HeLa cells that cholesterol can enter cells through other dynamin-independent endocytic pathways [9]. We next characterized dynasore in HMDM since they play a central role in the formation and progress of atherosclerotic plaques [22]. The uptake of AcLDL in HMDM was twice as much less efficient than the uptake of LDL in HeLa cells (Figure 1C). The effect of dynasore was less important in HMDM since the uptake of AcLDL uptake was decreased by 50% at 10 µg/ml and by 17% at 100 µg/ml. However, the absolute amount of endocytosed cholesterol was similar in HeLa cells and HMDM treated by dynasore. In agreement with the lower inhibition of AcLDL uptake, the measure of the total amount of cholesterol revealed no difference between control and dynasore-treated HMDM (Figure 1D).

### Dynasore blocks the egress of free cholesterol from the late endosomal network in HeLa cells and human macrophages

**Figure 2:**
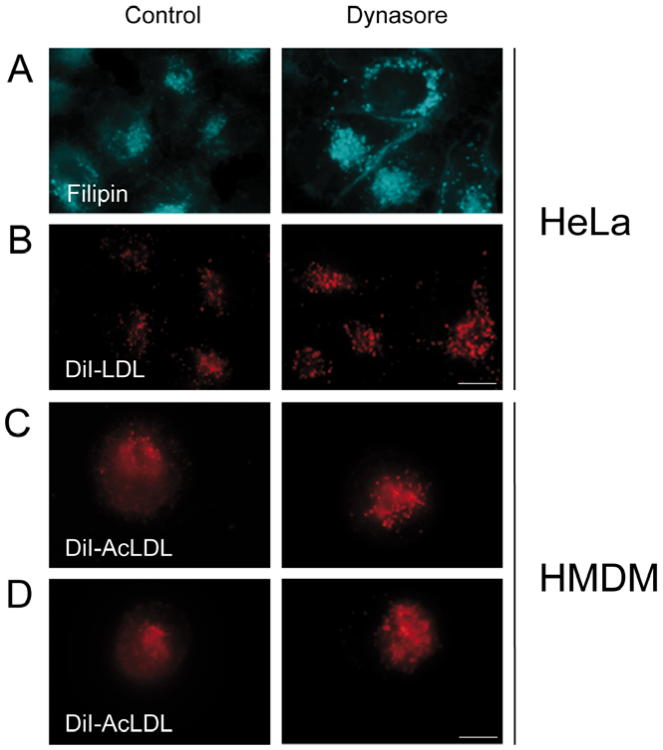
Effects of dynasore on the intracellular distribution of FC and LDL in HeLa cells and HMDM. (A) Hela cells were loaded with 200 µg/ml LDL for 24 h. Cells were then treated for 6 h with 80 µM dynasore or without (control) and stained with filipin to detect FC. (B) Cells were treated as described above with 200 µg/ml DiI-LDL. (C) HMDM were incubated for 6 h in LPDS medium containing 50 µg/ml DiI-AcLDL with 80 µM dynasore or without (control). (D) HMDM were loaded with 50 µg/ml DiI-AcLDL for 24 h and then treated for 6 h with 80 µM dynasore or without (control). Images were obtained using wide-field epifluorescence microscopy. Scale bars, 10 µm.

**Figure 3:**
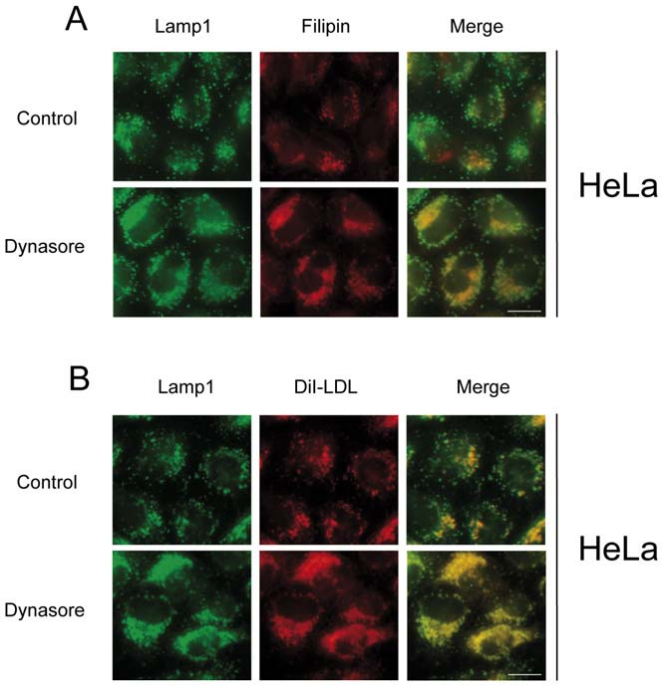
Dynasore treatment results in the endolysosomal accumulation of FC and LDL in HeLa cells. Cells were treated for 6 h with 80 µM dynasore or without (control) in medium containing 200 µg/ml LDL (A) or DiI-LDL (B) and processed for filipin staining (A) or DiI-LDL detection (B). Left panels present Lamp1 staining. Merge of Lamp1 with FC (A) or with DiI-LDL (B) is shown in the right panel. Scale bars, 10 µm.

We examined the intracellular distribution of FC derived from endocytosed LDL using filipin, a naturally fluorescent antibiotic that binds selectively to the FC fraction present in cellular membranes. We also visualized the intracellular distribution of DiI-LDL since we showed previously that DiI-LDL undergo the same endocytic and degradative processes than unlabeled LDL [9]. Cells were incubated with LDL for 24 hours to allow their distribution within the different endosomal compartments along the endocytic pathway. In control cells, FC was found into small endosomal structures within the cytoplasm of the cell (Figure 2A). A similar pattern was found for DiI-LDL (Figure 2B). Upon dynasore treatment, we could observe the accumulation of FC and Internalized DiI-LDL into numerous enlarged endosomes (Figures 2 A and B). We also examined the intracellular distribution of DiI-AcLDL in HMDM after 6 hours of internalization. Dynasore treatment led also to an increase of both the number and the size of the endosomal structures loaded with DiI-AcLDL (Figure 2C). This effect was enhanced when cells were first incubated with DiI-AcLDL for 24 hours before the addition of dynasore (Figure 2D). These endosomes were part of the late endosomal network since they were positive for the lysosomal associated membrane protein 1 (Lamp1), a marker of late endosomes and lysosomes (Figures 3 A and B). Thus, dynasore which blocks the GTPase activity of dynamin, causes the accumulation of endocytosed LDL-derived cholesterol in the late endocytic compartment and prevents its egress from this compartment in both HeLa and HMDM cells. This is in agreement with our previous study showing that dynamin controls the delivery of cholesterol from late endosomes to the ER in HeLa cells [9]. The abnormal endosomal accumulation of LDL was already observed after 15 min of dynasore treatment indicating that dynasore acts at the endoslysosomal level in the same order of time that it requires to inhibit the uptake of transferrin and LDL at the plasma membrane (data not shown) [10].

### Down-expression of dynamin results in the inhibition of free cholesterol egress from the late endosomal network

**Figure 4:**
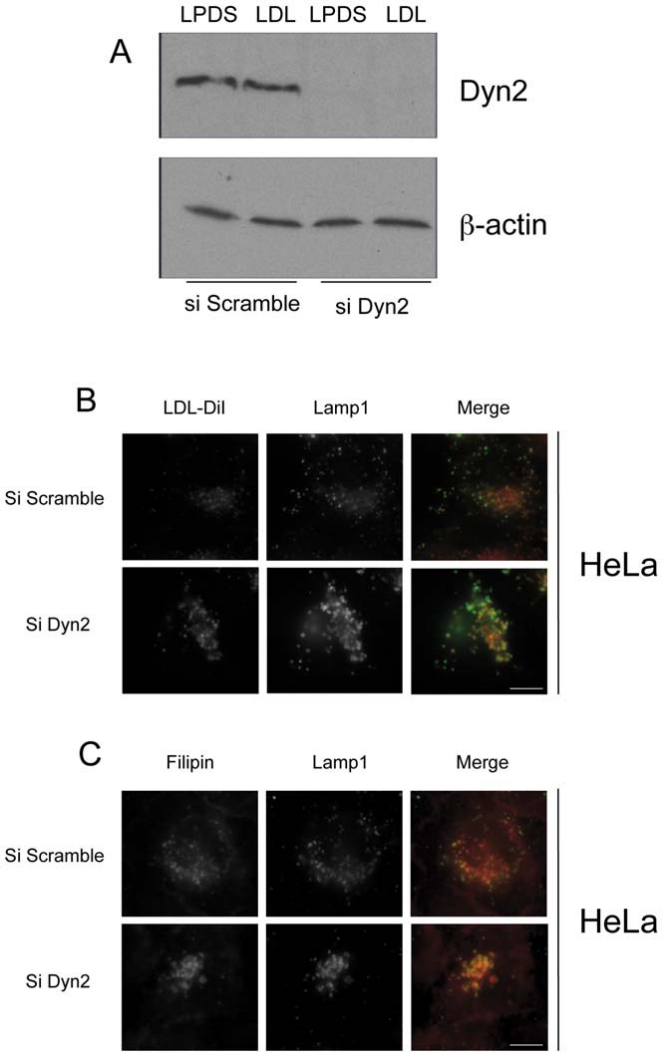
Dynamin silencing leads to endolysosomal accumulation of FC and LDL in HeLa cells. HeLa cells were transfected with Dyn2 siRNA or scramble siRNA (control) for 48 h in LPDS medium (LPDS) or LPDS medium and 200 µg/ml LDL (LDL). Dyn2 and actin levels were determined by western blot (A). Cells were loaded for 6 h with 200 µg/ml LDL and stained with filipin to detect FC (C). Cells were treated as described above with 200 µg/ml DiI-LDL (B). Images were obtained using wide-field epifluorescence microscopy. Scale bars, 10 µm.

To confirm the specificity of dynasore treatment, we depleted endogenous dynamin 2, the major dynamin isoform expressed in HeLa cells using specific siRNA. Dyn2 siRNa treatment led to a strong down-expression of dynamin 2 as assessed by real time quantitative RT-PCR (data not shown) and western blot analysis (Figure 4A). Under this treatment, there was an accumulation of swollen endosomal structures loaded with LDL or FC, and positive for Lamp1 (Figure 4 B and C). These results faithfully reproduce the phenotype observed with dynasore treatment and therefore exclude dynasore side effects at the endosomal interface.

### Dynasore prevents the down-regulation of sterol-sensitive genes induced by exogenous LDL-derived cholesterol

**Figure 5:**
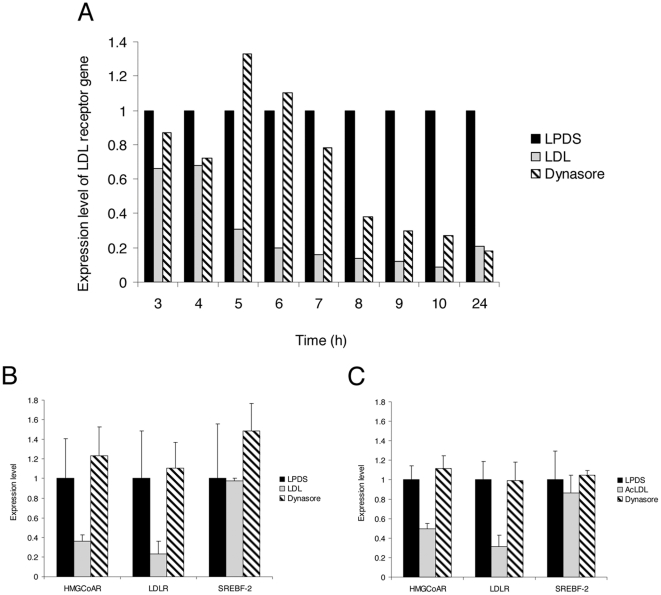
Dynasore blocks sterol-sensitive genes regulation in HeLa cells and HMDM. (A) Kinetics of LDLR expression analyzed by RT-PCR. HeLa cells were grown in LPDS medium for 48 h and further incubated for the indicated times with medium containing either LPDS, 200 µg/ml LDL, or 200 µg/ml LDL with 80 µM dynasore. (B) The expression level of sterol-sensitive genes (LDLR, HMGCoAR and SREBF-2) was quantified after 6 h in HeLa cells grown in LPDS, with 200 µg/ml LDL or 200 µg/ml LDL with 80 µM dynasore, as indicated. (C) The same experiment was performed in HMDM with 50 µg/ml AcLDL. Relative quantification of LDLR, HMGCoAR, and SREBF-2 genes in HeLa cells or HMDM was expressed as fold-variation over control (LPDS/DMSO) after normalization. All CT determinations were made in triplicate.

The delivery of FC from the late endolysosomal compartment to the ER is a key process in the transcriptional regulation of sterol-sensitive genes [1], [7], [9]. We therefore studied whether dynasore had an impact on this regulation. In contrast to the conditional K44A HeLa cell line that requires 48 hours to express the K44A dynamin mutant [9], dynasore is active within a few minutes. We thus determined the minimal amount of time required to measure an effect of dynasore on the expression of the LDLR gene, one of the major actors of the transcriptional control of cholesterol homeostasis [2]. After 48 hours of sterol starvation, cells were incubated with LDL in the presence or in the absence of dynasore. The kinetics of expression of the LDLR gene were monitored by real time quantitative RT-PCR analysis in HeLa cells (Figure 5A). As expected in cells that are in excess of exogenous cholesterol, a potent repression of the expression of the LDLR gene was measured as early as 5 hours after the addition of LDL and for as long as 24 hours in control cells. In contrast, no down-expression of the LDLR gene could be measured in cells treated with dynasore at similar times, and LDLR expression levels were similar to those observed in cells not supplemented with LDL. Seven hours after LDL addition, however, the effect of dynasore decreased progressively and the level of LDLR gene expression resumed to control values at 24 hours. Thus, we chose to analyze the effects of dynasore after 6 hours of LDL addition, which corresponds to a strong repression of the sterol-sensitive transcriptional response in control cells and to the maximal inhibition by dynasore. At 6 hours, dynasore also strongly inhibited the LDL-induced repression of another major sterol-sensitive gene, HMGCoAR, whereas the repression on SREBF-2 gene expression was not observed at this time in HeLa cells (Figure 5B). Similar results were obtained in dynasore-treated HMDM, with a strong inhibition of the down-regulation of sterol-sensitive genes (LDLR, HMGCoAR and to a lesser extent, SREBF-2) induced by AcLDL (Figure 5C). These data are in agreement with the accumulation of LDL and AcLDL in enlarged endosomes observed in both cell types (Figure 2) and are likely to reflect a defect in the delivery of LDL-derived cholesterol to the ER.

### Dynasore prevents the esterification of exogenous LDL-derived cholesterol

**Figure 6:**
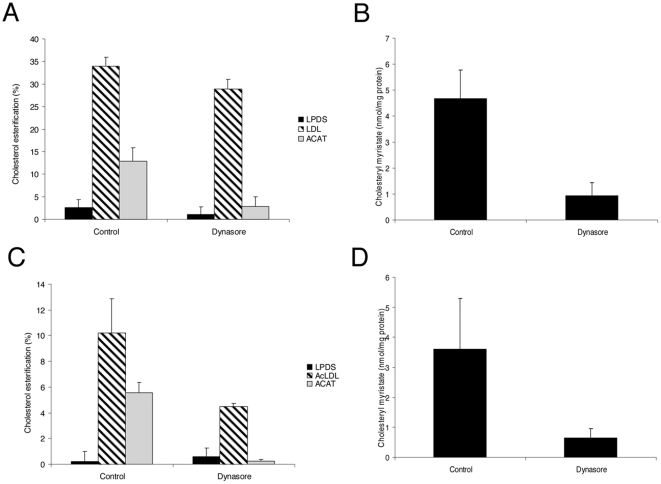
Dynasore decreases the production of cholesterol esters from LDL- or AcLDL-derived cholesterol. HeLa cells or HMDM were respectively incubated with 200 µg/ml LDL (A) or 50 µg/ml AcLDL (C) and treated for 6 h with 80 µM dynasore or without (control). The total amount of CE was quantified and expressed as the percent of the total amount of cholesterol. ACAT-dependent ester formation was measured with 10 µg/ml ACAT inhibitor (grey bars). The production of cholesteryl myristate was measured in HeLa cells (B) or HMDM (D) treated or not (control) with 80 µM dynasore. Cholesteryl myristate was expressed in nmol/mg protein. Each value is the mean of triplicate experiments.

We next measured the fraction of esterified cholesterol by the ACAT enzyme as a marker of the amount of FC being delivered to the ER. Indeed, CE are generated from FC by the activity of the ACAT enzyme [7]. Since this enzyme is strictly localized in the ER membranes, the amount of cholesterol esterified by ACAT reflects the amount of FC delivery to the ER [23], [24]. Thus, we measured by HPLC the balance between the pools of free and esterified intracellular cholesterol. When HeLa cells were grown under sterol starvation, cholesterol was mainly detected as FC and esters represented less than 5% of total cholesterol (Figure 6A). After the addition of LDL, the total amount of CE represented more than 30% of total cholesterol. Dynasore treatment reduced this amount by about 15%. This moderate inhibition disagrees with the complete absence of sterol-sensitive genes repression in dynasore treated cells as observed above. Thus, we studied whether a fraction of the measured pool of CE may be independent from the ER-ACAT activity. Indeed, dynasore treatment leads to the abnormal endosomal accumulation of LDL, which are unlikely to be de-esterified by the lysosomal hydrolases and thus could contribute to the total intracellular pool of CE. Therefore, we measured the total amount of CE in HeLa cells in which ACAT activity was pharmacologically inhibited. Under this condition, we found that the amount of CE generated by ACAT accounted for only 38% of the total intracellular pool of CE (Figure 6A). When cells were treated with dynasore, this amount decreased to about 10%, which represents a 74% inhibition of LDL-derived cholesterol esterification. We could confirm this result by measuring the synthesis of cholesteryl myristate by ACAT, an ester that was not initially present in our cells. After addition of myristate, we found by HPLC that the production of cholesteryl myristate was decreased by 80% in cells treated with dynasore (Figure 6B).

In HMDM, dynasore treatment decreased the total amount of CE more efficiently than in HeLa cells (Figure 6C). However, the level of CE was initially lower in HMDM than in HeLa cells (11% versus 35%, respectively). This could be due to different kinetics of AcLDL uptake and the lower concentration used (50 µg/ml). Indeed, after 24 hours of incubation with AcLDL, the level of CE in HMDM was higher and represented 40% of total cholesterol (data not shown). Again, dynasore treatment led to a strong decrease (90%) of the amount of CE specifically generated by ACAT (Figure 6C) and of the incorporation of myristate into CE (82% inhibition) (Figure 6D). Together these findings demonstrate that the abnormal endosomal accumulation of LDL induced by dynasore treatment results in a strong defect of FC delivery to the ER.

### Dynasore decreased cholesterol efflux in human macrophages

**Figure 7:**
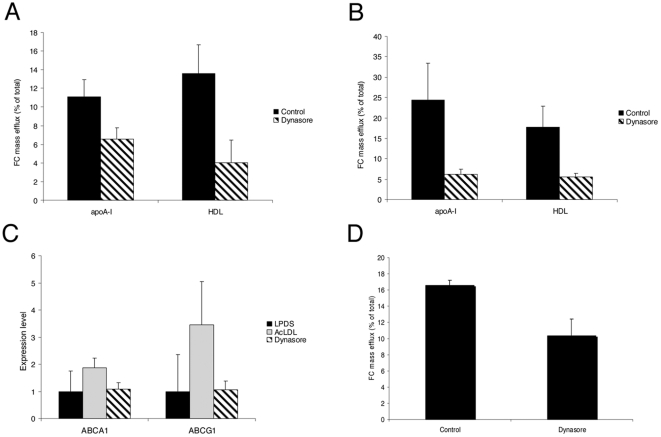
Dynasore impairs cellular cholesterol efflux from HMDM. HMDM were incubated for 6 h with 100 µg/ml AcLDL with 80 µM dynasore or without (control). The cellular cholesterol efflux to 10 µg/ml apoA-I or 15 µg/ml HDL-PL before (A) and after (B) stimulation of ABCA1 and ABCG1 expression by the LXR/RXR agonists was quantified. Results are expressed as the percentage of the quantity of released cellular cholesterol into the medium to the total quantity of cholesterol in cells and medium. Each value is the mean of triplicate experiments. (C) Relative quantification of ABCA1 and ABCG1 transporter genes levels was expressed as fold-variation over control (DMSO/LPDS) after normalization. All CT determinations were made in triplicate. (D) Passive cholesterol efflux to 1 mg/ml MâCD was quantified as above.

Although reverse cholesterol transport (RCT) is a general peripheral process, macrophages are the primary cells that are overloaded with cholesterol in atherosclerotic lesions [25]. The efficiency of cholesterol efflux, the first step of RCT from macrophage foam cells, is determined not only by the activity of cholesterol transporters and extracellular acceptors, but also by the availability of cholesterol for efflux. It was therefore interesting to test whether the abnormal sequestration of large amounts of cholesterol in the endolysosomal compartment could affect the cholesterol efflux capacity of HMDM. Thus, we measured cholesterol mass efflux to either lipid-free apoA-I or to HDL (high density lipoproteins) in HMDM loaded with AcLDL (Figure 7A). Dynasore treatment resulted in a strong decrease of cholesterol efflux to apoA-I (40%) and HDL (70%). Similar results were observed when cholesterol efflux was stimulated with the natural LXR/RXR agonists, 22OHC/9cRA (Figure 7B). The two ABC (ATP binding cassette) members of cholesterol transporters, ABCA1 and ABCG1 have been involved in cholesterol efflux to lipid poor apoA-I and HDL, respectively [25], [26]. We thus examined the impact of dynasore treatment on ABCA1 and ABCG1 gene expression. As expected, the addition of AcLDL to HMDM led to an increase of ABCA1 and ABCG1 mRNA levels (1.9-fold and 3.5-fold, respectively). However, this up-regulation was completely abolished when HMDM were treated by dynasore (Figure 7C). Finally, we examined whether dynasore treatment could affect the amount of cholesterol associated with the plasma membrane, which is involved in the passive cholesterol efflux pathway. To address this question, we used MβCD, a high-affinity acceptor of cholesterol. Short time incubation with MβCD was shown to remove cholesterol from the plasma membrane [27]. Under these conditions, cholesterol efflux was significantly decreased (34%) in dynasore-treated cells (Figure 7D). Together, these results indicate that dynasore, by affecting intracellular cholesterol trafficking, has a major impact on several pathways implied in active and passive cholesterol efflux.

### Dynasore affects the intracellular distribution of the mannose 6-phosphate receptor

**Figure 8:**
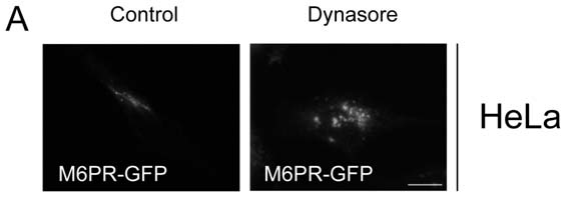
Dynasore treatment affects M6PR distribution. (A) Cells expressing GFP-M6PR were treated 6 h with 80 µM dynasore or without (control). Images were obtained using wide-field epifluorescence microscopy. Scale bars, 10 µm.

We tested whether dynasore could affect the trafficking of other cargos through the late endosomal network. We therefore studied the cation-independent mannose 6-phosphate receptor (M6PR), which constitutively recycles between late endosomes and the Golgi complex [28]. When HeLa cells expressing a GFP tagged M6PR were treated by dynasore, we observed that the M6PR was not present at the Golgi complex and was localized instead in dispersed endosomal structures (Figure 8). This result, in agreement with a previous study [28], indicates that the effect of dynasore was not restricted to the block of FC from the late endosomal network but affected also cargo trafficking at this interface.

### Comparison of dynasore and U18666A treatments on cholesterol trafficking in HeLa cells and macrophages

**Figure 9:**
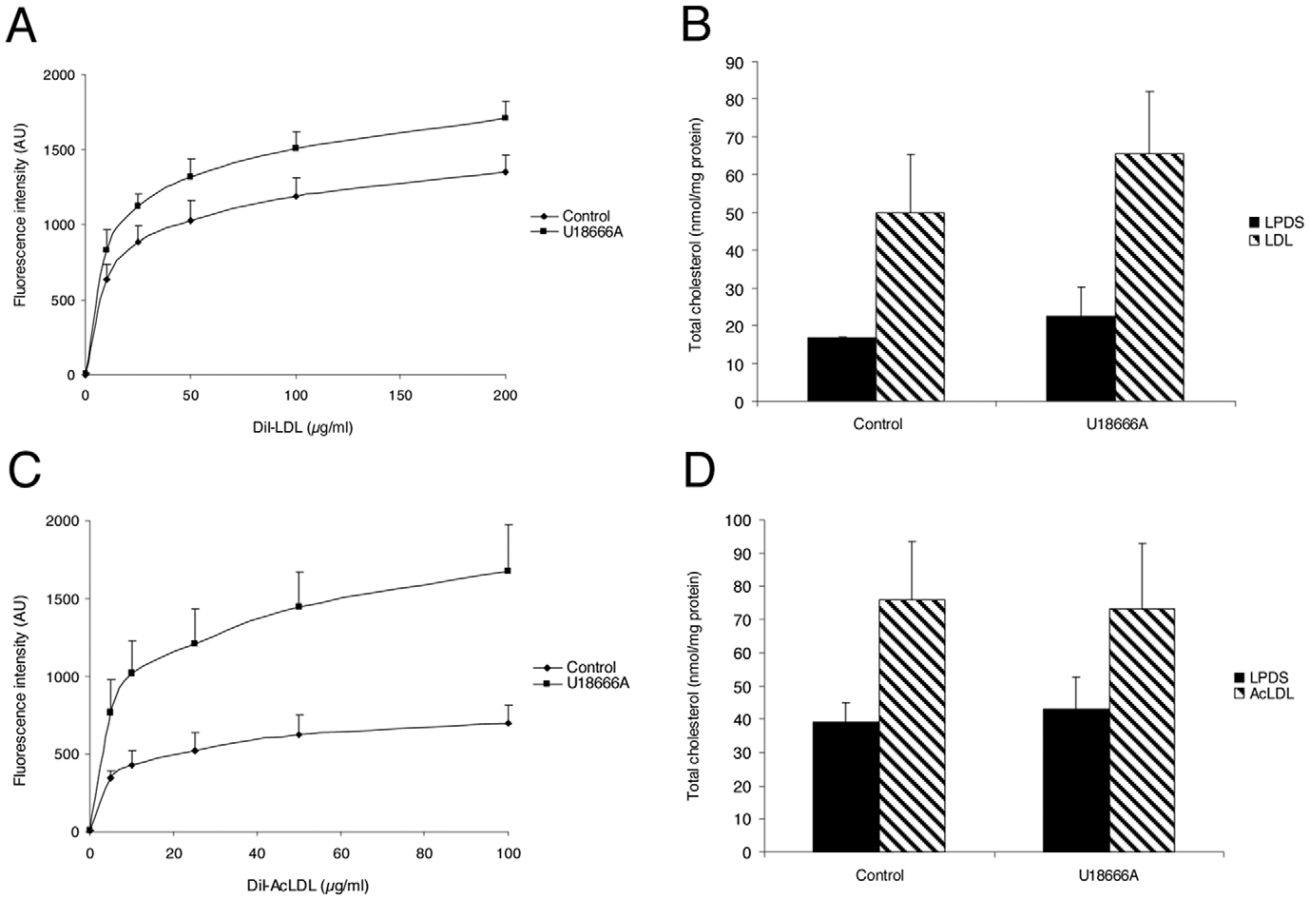
Effect of U18666A on LDL uptake and total cholesterol in HeLa cells and HMDM. LDL uptake was measured in HeLa cells (A) and HMDM (C) after incubation at 37°C for 4 h with 0–200 µg/ml DiI-LDL or 0–100 µg/ml DiI-AcLDL, respectively, with 3 µg/ml U18666A or without (control). The amount of endocytosed DiI-LDL and DiI-AcLDL was measured by flow cytometry. Values represent the mean ± SD of triplicate experiments. Total cholesterol was quantified in HeLa cells (B) and HMDM (D) after 4 h of LDL uptake with 3 µg/ml U18666A or without (control). Each value is the mean ± SD of triplicate experiments and expressed as nanomoles per mg of cell proteins.

**Figure 10:**
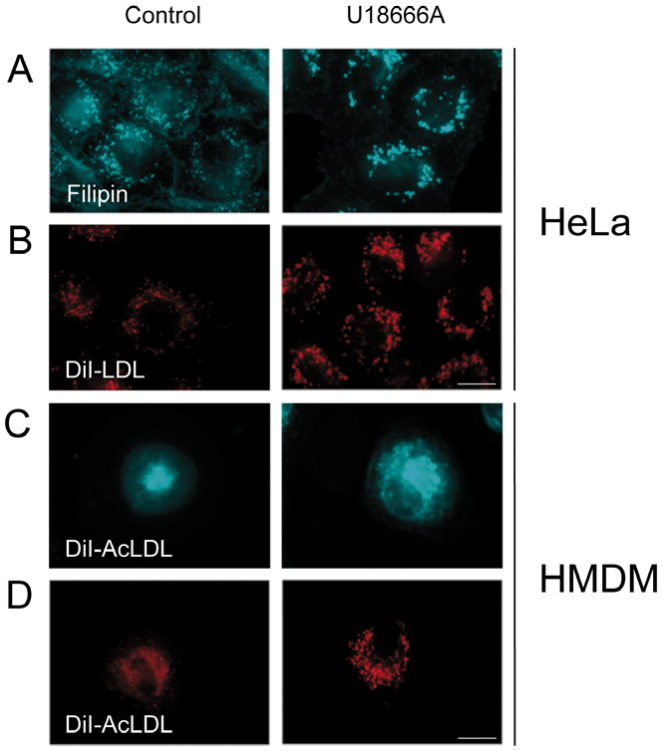
Effects of U18666A on the intracellular distribution of FC and LDL in HeLa cells and HMDM. HeLa cells and HMDM were respectively incubated for 6 h with 200 µg/mL LDL (A) or 50 µg/ml AcLDL (C) with 3 µg/ml U18666A or without (control) and stained with filipin to detect FC. (B–D) HeLa cells and HMDM were respectively incubated for 6 h with 200 µg/ml DiI-LDL (B) or 50 µg/ml DiI-AcLDL (D) with 3 µg/ml U18666A or without (control) and processed to visualize LDL distribution. Images were obtained using wide-field epifluorescence microscopy.

**Figure 11:**
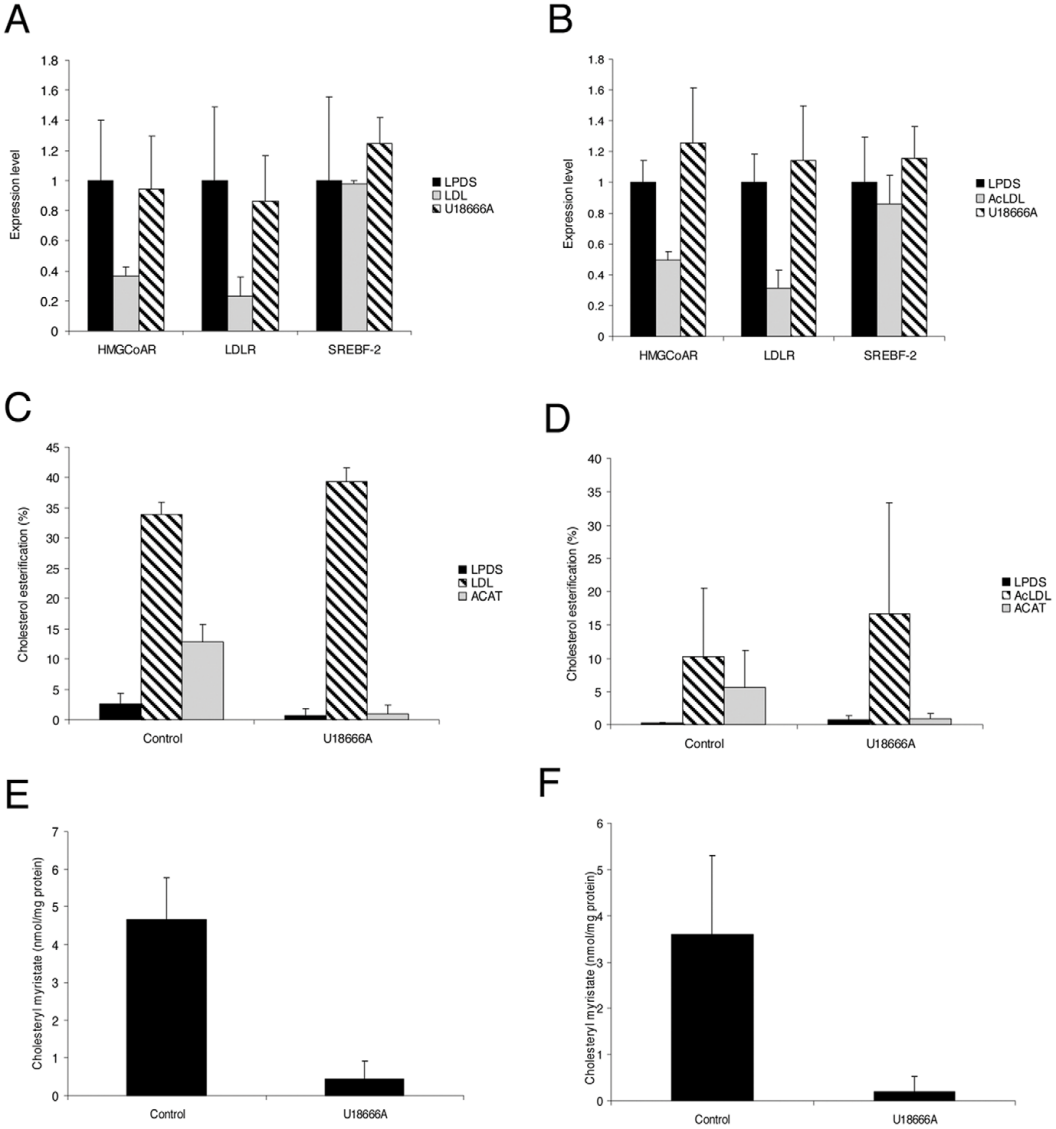
U18666A inhibits ACAT activity and sterol-sensitive genes regulation in HeLa cells and HMDM. Cells were grown in LPDS medium for 48 h and further incubated for 6 h with 200 µg/ml LDL (A) or 50 µg/ml AcLDL (B) with 3 µg/ml U18666A or without (control). Relative quantification of LDLR, HMGCoAR, and SREBF-2 genes in HeLa cells (A) or HMDM (B) was expressed as fold-variation over control (LPDS/DMSO) after normalization. All CT determinations were made in triplicate. The total amount of CE was quantified HeLa cells (C) and in HMDM (D) and expressed as the percent of the total amount of cholesterol. ACAT-dependent ester formation was measured with 10 µg/ml ACAT inhibitor (grey bars). Cholesteryl myristate formation was measured in HeLa cells (E) or HMDM (F) with 3 µg/ml U18666A or without (control). Cholesteryl myristate was expressed in nmol/mg protein. Each value is the mean of triplicate experiments.

These results led us to compare the effects of dynasore with U18666A, a hydrophobic amine that has been extensively used to study cholesterol homeostasis, and more particularly cholesterol intracellular trafficking [29]. In contrast to dynasore, we found that U18666A treatment led to a respective 20% and 40% increase of LDL uptake and AcLDL in HeLa cells and HMDM (Figure 9 A and C). Accordingly, we measured an increase in the total amount of cholesterol present in HeLa cells and HMDM after U18666A treatment (Figures 9 B and D). The inhibition of cholesterol egress from late endosomes and lysosomes is a well-known effect of U18666A [29], [30]. As expected, treatment with U18666A led to the formation of swollen endosomal structures loaded with FC in HeLa cells and HMDM (Figure 10). These structures were part of the endolysosomal network as confirmed by staining with Lamp1 (not shown). As a consequence, there was no response of sterol-sensitive genes to the addition of LDL or AcLDL in U18666A treated cells (Figures 11 A and B). In contrast to cells treated with dynasore, we observed a slight increase in the percentage of CE in U18666A-treated cells (Figures 11 C and D). The measure of CE in cells loaded with cholesterol in the presence the ACAT inhibitor revealed that the fraction of CE specifically generated by ACAT was 54% of the total intracellular pool of CE. When cells were treated with U18666A, the amount of CE generated by ACAT was decreased by 90%. Likewise the incorporation of myristate into CE was strongly inhibited by U18666A (Figure 11 E and F).

**Figure 12:**
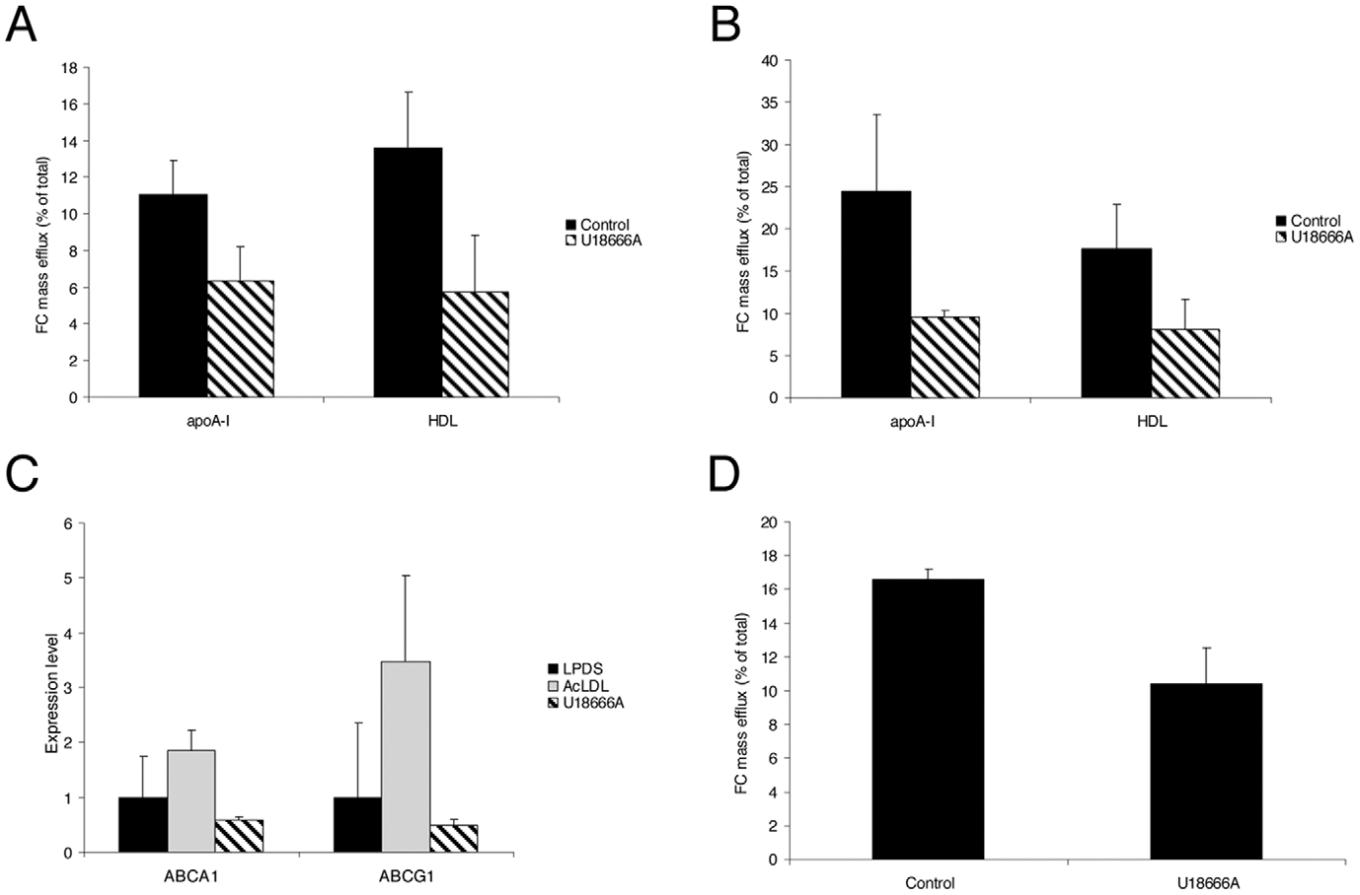
U18666A impairs cellular cholesterol efflux from HMDM. Cells were incubated with 100 µg/ml AcLDL for 6 h and treated with 3 µg/ml U18666A or without (control). The cellular cholesterol efflux to 10 µg/ml apoA-I or 15 µg/ml HDL-PL before (A) and after (B) stimulation of ABCA1 and ABCG1 expression by the LXR/RXR agonists was quantified. Results are expressed as the percentage of the quantity of released cellular cholesterol into the medium to the total quantity of cholesterol in cells and medium. Each value is the mean of triplicate experiments. (C) Relative quantification of ABCA1 and ABCG1 transporter genes levels expressed as fold-variation over control (DMSO/LPDS) after normalization. All CT determinations were made in triplicate. (D) Passive cholesterol efflux to 1 mg/ml MâCD was quantified as above.

Finally, we also measured the effects of U18666A on cholesterol efflux. As shown in Figures 12 A and D, the cellular cholesterol efflux to apoA-I, HDL, and MβCD was decreased by 42%, 58%, and 37%, respectively when macrophages were treated with U18666A. A similar decrease was measured in AcLDL-loaded macrophages after stimulation by the LXR/RXR agonists, 22OHC/9cRA (Figure 12B). Like dynasore, U18666A completely blocked the increase of ABCA1 and ABCG1 mRNA levels in response to the addition of AcLDL in HMDM (Figure 12C).

## Discussion

Dynasore is a small drug that was identified through a chemical screen designed for inhibitors of the dynamin GTPase activity [10]. Because of its cell membrane permeability and reversibility, dynasore has since been extensively used to block in a selective and powerful manner the different endocytic pathways that rely on the dynamin GTPase. In a previous work based on the expression of the K44A inactivated mutated form of dynamin, we could demonstrate that dynamin activity was also required for proper sorting of cholesterol in the endolysosomal network of HeLa cells. K44A dynamin expression led to the inhibition of free cholesterol endosomal egress to the ER, which instead accumulated into swollen endosomes of the endolysosomal network. As a result, the sterol-sensitive gene regulation was inhibited [9].

In this study, we took advantage of the cell permeability of dynasore to further extend our understanding of cholesterol intracellular trafficking in human macrophages. We chose to work on HMDM isolated from peripheral blood since available mouse or human macrophages cell lines do not entirely reproduce the characteristics of primary cultured HMDM. The monocytes were differentiated into macrophages by hM-CSF instead of hGM-CSF since it favors the expression of cell surface markers that are closer to those found in macrophages from atherosclerotic lesions [31]. AcLDL were used instead of native LDL since AcLDL uptake is 20-fold higher in human macrophages [32]. Indeed, the class A macrophage scavenger receptor SR-A accounts for the majority of AcLDL uptake, a pathway that is unregulated and can lead to the intracellular accumulation of large amounts of cholesterol and foam cell formation. In contrast, the uptake of native LDL is minor and regulated in macrophages [33]. Moreover, Wang *et al.* have shown in macrophages that AcLDL-delivered cholesterol is preferentially transported into the late endosomal network whereas LDL-derived cholesterol is preferentially transported to the recycling compartment [34]. In this study, we show that dynasore recapitulates the effects of the K44A dynamin mutant on cholesterol homeostasis that we have first described in HeLa cells. Dynasore, however, was more efficient to block LDL uptake than the K44A mutant, which probably reflects the more homogenous cell distribution of the drug. Interestingly, the inhibition of AcLDL uptake by dynasore was less efficient in human macrophages. This is in agreement with the lesser inhibition of AcLDL uptake that was also reported in HMDM after down-expression of clathrin [35]. Macrophages can use several alternative pathways such as macropinocytosis or caveolae for the uptake of AcLDL [35], [36]. Our data suggest that the dynamin-independent endocytic pathways are the main contributors to AcLDL uptake in HMDM. In contrast to dynasore, U18666A leads to an increase of LDL uptake in both HeLa cells and HMDM. This increase, which is much higher in HMDM cells, can be inhibited by dynasore (not shown) indicating that U18666A up-regulates LDL uptake through the classical endocytic pathways. The increased LDL uptake is probably due to the known increased expression of LDL receptors resulting from the inhibition of FC delivery to the ER by U18666A. Dynasore treatment, which also leads to an increase of LDL receptor gene expression (Figure 4), does not result however in increased LDL uptake, because of its inhibitory effect on LDLR endocytosis through clathrin-coated pits.

We show that dynasore blocks the delivery of exogenous LDL-derived cholesterol from the endolysosomal network to the ER, resulting in the inhibition of both sterol-sensitive genes regulation and cholesterol esterification. Similar findings were found in cells where dynamin 2 was down-expressed by RNAi treatment confirming the specificity of dynasore effect at the late endosomal network. Interestingly, dynasore and U18666A present the same effects on cholesterol membrane trafficking and sterol-sensitive genes regulation.

The kinetics of the inhibition of LDL induced sterol-sensitive genes down-regulation showed that dynasore activity was maximal at 6 hours. We observed a progressive decrease of dynasore effect with time, an effect not observed with U18666A. Whether this is due to an inactivation of the drug with time or to the delivery of FC to the ER by alternative pathways has to be documented. In this context, it is interesting that the inhibition of LDL uptake by dynasore persisted for 24 hours suggesting that the drug was still active at this time (data not shown).

Different cholesterol efflux pathways have been described that occur either by a non regulated diffusive process, or actively mediated by SR-BI and ABC transporters [26]. Recent studies indicate that ABCA1 and ABCG1 are the main contributors to the net cholesterol efflux from macrophages to HDL or serum [34], [37], [38]. Our data show that the cholesterol efflux mediated by ABCA1 and ABCG1 was impaired in dynasore-treated HMDM, even after stimulation with the LXR/RXR agonist. Similar results were obtained with U18666A. It has been shown that the endolysomal sequestration of LDL-derived cholesterol in NPC^−/−^ cells altered cholesterol efflux by reducing the amount of cholesterol substrate available for ABCA1 and by defective synthesis of LDL-cholesterol-derived side-chain oxysterols [39], [40]. The decreased production of oxysterols, which are endogenous LXR ligands, results in reduced ABCA1 and ABCG1 expression and lower cholesterol efflux activity [41]. 27 hydroxycholesterol is the most abundant oxysterol present in macrophages. A key step in 27 hydroxycholesterol synthesis is the delivery of cholesterol to mitochondria where is present the sterol-27 hydroxylase CYP27. It is therefore tempting to speculate that the decrease of ABCA1 and ABCG1 gene expression is due to a decreased delivery of cholesterol to mitochondria. Alternatively, the decrease of cholesterol efflux mediated by ABCA1 and ABCG1 could be the consequence of a reduction of the pool of FC at the plasma membrane since it is a preferential site to collect FC coming from the endolysosomal compartment.

The role of dynamin in endolysosomal sorting remains poorly documented. We show here that dynasore treatment affects not only cholesterol trafficking but also the intracellular distribution of the M6PR. A recent work has shown that dynamin 2 controlled the exit of the EGFR from late endosomes through its association with CIN85 [42]. Altogether these data demonstrate that dynamin controls trafficking events within the endolysosomal system presumably through the scission of vesicular buds originating from early and/or late endosomes and affects several cargos that use this pathway.

In conclusion, we have shown that the pharmacological inhibition of the dynamin GTPase activity by dynasore leads rapidly to the abnormal endosomal sequestration of FC and LDL, resulting in defective sterol-sensitive genes regulation and cholesterol efflux in HMDM. These results demonstrate that dynasore can be used to block the egress of FC from the endolysosomal network. Dynasore therefore represents an interesting alternative to U18666A and will be useful to better understand the complexity of cholesterol trafficking and homeostasis at the late endosomal interface.
